# Andrographolide suppresses chondrosarcoma cell migration and invasion by inhibiting the PI3K/Akt/mTOR signaling pathway and activating autophagy

**DOI:** 10.3389/fonc.2025.1622666

**Published:** 2025-09-26

**Authors:** Chun-Fei Wu, Li-xin Ouyang, Wan-hao Zhang, Shuo Yang, Xian-Xing Zhong, Yong Lin, Shuai Fan, Peng Li, Zu-Jian Liang

**Affiliations:** ^1^ The Third Affiliated Hospital of Guangzhou University of Chinese Medicine, Guangzhou, China; ^2^ Guangdong Research Institute for Orthopedics and Traumatology of Chinese Medicine, Guangzhou, China; ^3^ The Fourth Clinical Medical College of Guangzhou University of Chinese Medicine, Shenzhen, China; ^4^ The Second Clinical College of Guangzhou University of Chinese Medicine, Guangzhou, China; ^5^ Shenzhen Traditional Chinese Medicine Hospital, Shenzhen, China

**Keywords:** andrographolide, chondrosarcoma, PI3K/AKT/mTOR, network pharmacology, *in vitro* experiments

## Abstract

**Objective:**

Chondrosarcoma, a malignant bone tumor, exhibits a high incidence rate. This study employed network pharmacology and cell-based experiments to explore the molecular mechanisms by which andrographolide (Andro) suppressed the migration and invasion of chondrosarcoma cells.

**Methods:**

Andro’s target genes were identified through integration of data from SuperPred, SEA, STITCH, Pharmmapper, HERB, HIT-2, and Swiss Target Prediction databases, and subsequently cross-referenced with chondrosarcoma-related genes. A protein–protein interaction (PPI) network was constructed using the STRING platform, followed by GO functional annotation and KEGG pathway enrichment analyses of potential targets with R software. Molecular docking assessed the binding affinities between Andro and key targets. Based on network pharmacology data, *in vitro* experiments validated Andro’s impact on the migration and invasion of chondrosarcoma cells and investigated its underlying mechanisms.

**Results:**

A total of 167 potential targets for Andro were identified. The PPI network highlighted PI3K, Akt, and mTOR as core targets. KEGG pathway analysis revealed that Andro’s inhibitory effects on cell migration and invasion were linked to the PI3K/Akt, HIF-1, and T-cell receptor signaling pathways. Molecular docking confirmed that the binding energies for the Andro-PI3K, Andro-Akt, and Andro-mTOR complexes were<–5 kcal/mol. Wound healing and transwell assays demonstrated that Andro (5 and 20 μM) treatment significantly reduced the wound healing rate and impaired the migratory and invasive abilities of chondrosarcoma cells after 24 hours (*p<*0.05) compared to controls. Western blotting (WB) analysis showed that Andro (5, 20, and 50 μM) notably downregulated vimentin and MMP-9 expression while upregulating E-cadherin in chondrosarcoma cells (*p<*0.05 for all). Furthermore, Andro (5, 20, and 50 μM) decreased the p-mTOR/mTOR, p-PI3K/PI3K, and p-Akt/Akt ratios in SW1353 and Hs 819.T cells. WB results also revealed that Andro (5, 20, and 50 μM) reduced p62 expression, while Beclin-1 expression and the LC3A/B-II/LC3A/B-I ratio increased in SW1353 and Hs 819.T cells. Confocal microscopy demonstrated a significant increase in autophagic flux in Andro-treated SW1353 cells. Andro’s effects were attenuated by autophagy inhibitor chloroquine, indicating its pharmacological action via autophagy inhibition in chondrosarcoma cells.

**Conclusion:**

Therefore, Andro could reduce the migration and invasion of chondrosarcoma cells by modulating the PI3K/Akt/mTOR signaling pathway, alleviating autophagy inhibition, and subsequently promoting autophagic activity.

## 1 Introduction

Chondrosarcoma represents a malignant bone neoplasm, comprising 20%–30% of all such tumors (1, 2). It affects individuals across all age groups, with a higher prevalence in men (1, 2). The pelvis and long bones, particularly the femur, are the most frequent locations for this tumor (3). Surgical resection remains the standard treatment, with tumor margin excision correlating with improved postoperative survival (3, 4). In contrast, chondrosarcoma exhibits resistance to radiotherapy and chemotherapy (5, 6), primarily due to factors such as limited angiogenesis, an abundant extracellular matrix, restricted cellular proliferation, and slow growth rates (1, 7). The anatomical features and invasive properties of common chondrosarcoma sites complicate effective medical intervention. Furthermore, surgical resection is often not a viable option for patients with metastatic chondrosarcoma (8, 9). Consequently, there is a pressing need for more targeted and effective pharmacological therapies for managing chondrosarcoma.

Andrographolide (Andro), a diterpenoid lactone with the molecular formula C_20_H_30_O_5_, is the principal bioactive compound derived from *Andrographis paniculata* (Burm. f.) Nees, a member of the Oleaceae family. Its molecular structure comprises three hydroxyl groups and a lactone ring (10, 11). Andro exhibits a range of biological activities, including anti-cancer, anti-inflammatory, antioxidant, and immune-modulatory effects (10, 11). According to Attri et al., Andro holds promise as a natural anti-cancer agent, capable of overcoming certain limitations inherent in conventional cancer treatments due to its broad-spectrum efficacy and low toxicity (12). The compound exerts anti-cancer actions by disrupting the cell cycle, suppressing cell migration, and inducing apoptosis and autophagic cell death at distinct biological stages (13–15). A previous investigation emphasized Andro’s role as an antiviral agent (16), exploring its activity and that of its derivatives against COVID-19 through systems biology, suggesting its involvement in various biological processes. Furthermore, Wang et al. demonstrated that Andro promoted protective autophagy by targeting DJ-1, triggering reactive oxygen species to induce pancreatic cancer cell death (17). This suggests that autophagy induction is a central mechanism behind Andro’s anti-cancer effects (17). Additionally, previous work has shown that Andro inhibits chondrosarcoma cell proliferation (18), though the mechanisms through which it prevents chondrosarcoma metastasis remain unclear.

**Figure 1:**
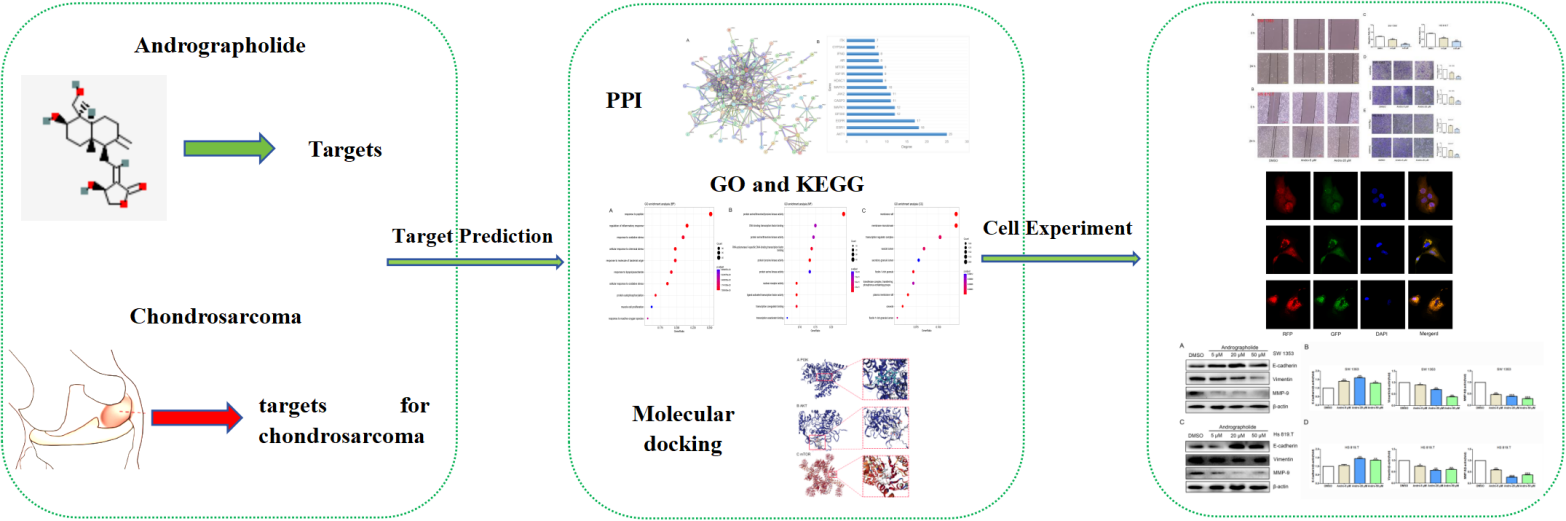
Study design.

The hypothesis that Andro suppresses the migratory and invasive potential of chondrosarcoma cells, with Andro-induced autophagy as a central mechanism, is derived from prior studies. This hypothesis was tested using network pharmacology and *in vitro* cell experiments to evaluate Andro’s effects on chondrosarcoma cell migration and invasion, as well as to identify the associated mechanisms (**Figure 1**).

## 2 Materials and methods

### 2.1 Network pharmacology

#### 2.1.1 Potential targets of Andro

The structural data for Andro were sourced from PubChem (https://pubchem.ncbi.nlm.nih.gov/), and potential targets were identified via SuperPred (http://prediction.charite.de/), SEA (http://sea.bkslab.org/), STITCH (https://ngdc.cncb.ac.cn/databasecommons/database/id/208), Pharmmapper (http://lilab-ecust.cn/pharmmapper/index.html), HERB (http://herb.ac.cn/), HIT-2 (http://www.badd-cao.net:2345/), and Swiss Target Prediction (http://www.swisstargetprediction.ch/) databases. Gene names were standardized using the UniProt database (https://www.uniprot.org/).

#### 2.1.2 Acquisition of chondrosarcoma-related genes

Chondrosarcoma-related genes were retrieved from Genecards (https://www.genecards.org/), the Comparative Toxicology Database (CTD) (http://ctdbase.org/), and MalaCards (https://www.malacards.org/) by searching for “Chondrosarcoma”. Intersection analysis was performed by comparing these genes with Andro’s target genes using a Venn diagram, identifying the overlapping genes as potential therapeutic targets of Andro in chondrosarcoma. The Venn diagram was generated with Venny (version 2.1.0) (https://bioinfogp.cnb.csic.es/tools/venny/index.html).

#### 2.1.3 Gene Ontology and Kyoto Encyclopedia of genes and genomes analyses

The clusterProfiler package in R (version 4.3.1) (http://www.R-project.org, The R Foundation) was utilized for Gene Ontology (GO) functional annotation and Kyoto Encyclopedia of Genes and Genomes (KEGG) pathway enrichment analyses of potential targets (19). GO analysis identified molecular functions (MFs), biological processes (BPs), and cellular components (CCs) linked to these targets, while KEGG analysis highlighted signaling pathways involved in the anti-cancer effects of Andro on chondrosarcoma. By applying a *P*-value threshold of <0.05 and a *Q*-value threshold of <0.05, key genes and pathways in the enrichment were identified.

#### 2.1.4 Construction of a protein–protein interaction network and identification of core targets

The therapeutic targets of Andro for osteosarcoma (identified in section 2.1.2) were analyzed using the STRING database. Data analysis parameters included specifying “*Homo sapiens*” as the species and setting the confidence threshold at ≥0.90. The resulting protein–protein interaction (PPI) network was imported into Cytoscape (version 3.7.1) for visualization. To identify key genes, the cytoHubba plugin, utilizing the Degree algorithm, was employed to rank the top 15 genes based on connectivity, with darker red shades representing higher scores. These 15 genes were designated as core targets for Andro’s therapeutic effect on chondrosarcoma.

#### 2.1.5 Molecular docking

The three-dimensional structures of the core target proteins and Andro were retrieved from the RCSB Protein Data Bank (RCSB PDB) (http://www.rcsb.org/). Following retrieval, structural preprocessing was conducted: missing atoms were repaired, hydrogen atoms were incorporated to preserve structural integrity, water molecules and other heteroatoms were removed to minimize interference, and energy minimization was performed to optimize the structures. Binding sites were identified using the protein pocket detection function of the CB-Dock2 platform, along with solvent-accessible surface area and electrostatic potential maps. Docking parameters were set according to the characteristics of the active sites. Molecular docking procedures and visualizations were carried out on the CB-Dock2 platform (20). A binding energy ≤-5 kcal/mol indicated favorable binding activity (21). Finally, *in vitro* experiments were performed to confirm the molecular docking results, with a focus on Andro’s regulatory effects on the core targets.

### 2.2 Experimental verification

#### 2.2.1 Cell culture and reagents

The human chondrosarcoma cell lines SW1353 and Hs 819.T (18) were acquired from the American Type Culture Collection (ATCC, Manassas, VA) and Beina Chuanglian Biotechnology Research Institute (Beijing, China), respectively. All cells were provided by the Institute of Orthopedic Diseases and the Center for Joint Surgery and Sports Medicine, the First Affiliated Hospital of Jinan University (18). Cells were cultured in high-glucose DMEM (Gibco) supplemented with 10% fetal bovine serum (FBS, Gibco) and 100 units/mL of penicillin/streptomycin (Gibco). Cultures were maintained in a humidified incubator with 5% CO_2_ at 37°C, with subculturing performed every 3 days. Andro (Sigma, MO, USA) was dissolved in dimethyl sulfoxide (DMSO). In all cell experiments, cells treated with an equivalent volume of DMSO (vehicle control) were included to account for any potential solvent-related effects. The concentration of drug intervention was determined using the CCK-8 assay kit (Beyotime Biotechnology, Shanghai, China).

#### 2.2.2 Wound healing assay

SW1353 and Hs 819.T cells were seeded into 6-well plates and cultured until a confluent monolayer was established. Following medium removal, a sterile 1000-μL pipette tip was used to create a scratch at the center of the monolayer. The cells were subsequently washed with PBS to eliminate non-adherent cells. After 24 hours of treatment with varying concentrations of Andro (5 and 20 μM) (18), wound healing was assessed by observing the cells under a microscope, and images were captured to evaluate the healing rate.

#### 2.2.3 Transwell migration assay

A Transwell insert (8 μm, 353097, BD) was positioned in a 24-well plate. A 200 μL suspension of SW1353 or HS 819.T cells (2×10^4^ cells) was added to the upper chamber, while 500 μL of culture medium supplemented with 10% FBS was added to the lower chamber. The cells were exposed to various concentrations of Andro (5 and 20 μM) and incubated for 24 hours. Afterward, non-migratory cells on the upper surface of the insert membrane were removed with a cotton swab, and cells that had migrated to the lower surface were fixed with pre-cooled methanol for 30 minutes and stained with 0.3% crystal violet (C0121, Beyotime) for 3 minutes. Following this, the cells were washed with PBS, and the number of migrated cells was counted under a microscope.

#### 2.2.4 Transwell invasion assay

An 8-μm Transwell insert was pre-coated with 100 μL of Matrigel Matrix (356231, BD). Once the Matrigel solidified, 200 μL of SW1353 or Hs 819.T cell suspension (2×10^4^ cells) was added to the upper chamber. The following procedures mirrored those of the transwell migration assay.

#### 2.2.5 Western blotting

After 24 hours of treatment with Andro at concentrations of 5, 20, and 50 μM, both adherent and non-adherent cells were harvested for protein expression analysis via western blotting (WB) as previously described (22). The following primary antibodies were utilized for WB: anti-β-actin (1:2000, 3700, CST), anti-E-cadherin (1:1000, 14472, CST), anti-vimentin (1:1000, 5741, CST), anti-MMP-9 (1:1000, ab76003, abcam), anti-mTOR (1:1000, 2983, CST), anti-p-mTOR (Ser2448) (1:1000, 5536, CST), anti-PI3K (1:1000, 3358, CST), anti-PI3K p85 (1:1000, ab182651, abcam), anti-Akt (1:1000, 4691, CST), anti-phospho-Akt (Ser473) (1:1000, 4058, CST), anti-p62 (1:5000, ab109012, Abcam), anti-Beclin1 (1:1000, 3459, CST), and anti-LC3A/B (1:1000, 12741, CST).

#### 2.2.6 Immunofluorescence

Immunofluorescence was performed using the following reagents: mRFP-GFP-LC3 lentivirus (GeneChem, China) and DAPI. SW1353 cells were infected with the mRFP-GFP-LC3 lentivirus, and the infected cells were subsequently plated in 24-well plates containing coverslips. After a 24-hour incubation period, cells were treated with Andro at concentrations of 20 μM and 50 μM for an additional 24 hours. Following treatment, DAPI solution was applied for nuclear staining prior to immunofluorescence analysis.

#### 2.2.7 Statistical analysis

Cell experiments were conducted with three independent biological replicates. Data were analyzed using one-way ANOVA in GraphPad Prism 8.0 software and presented as the mean ± standard error of the mean (SEM). A *p*-value <0.05 was considered statistically significant, with asterisks indicating the level of significance: *, *p<*0.05; **, *p<*0.01; ***, *p<*0.001.

## 3 Results

### 3.1 Potential targets of Andro for the treatment of osteosarcoma

**Figure 2:**
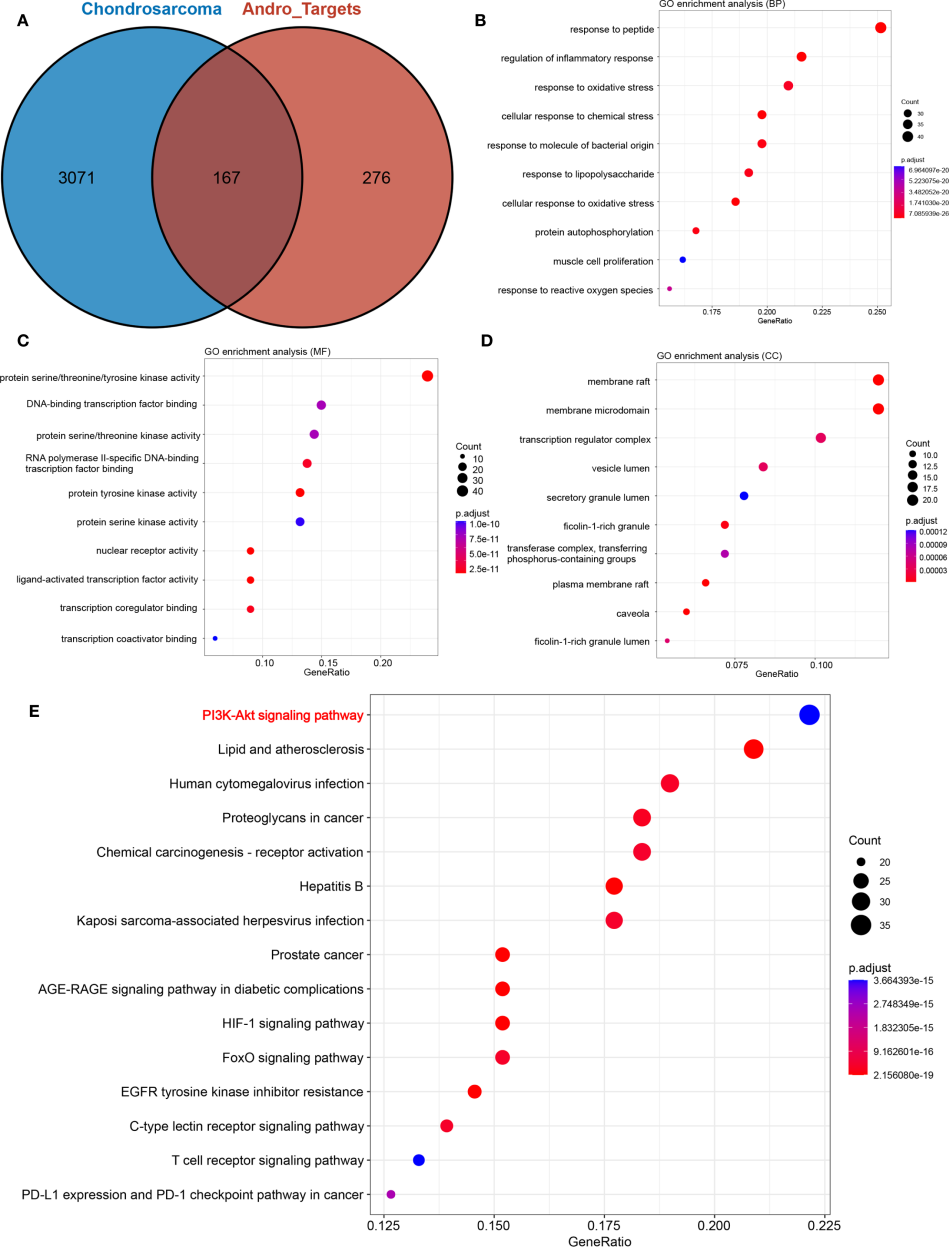
**(A)** Venn diagram showing the intersection of target genes of andrographolide with chondrosarcoma-related genes; **(B)** biological processes (BP); **(C)** cellular components (CC); **(D)** molecular functions (MF); **(E)** Bubble chart demonstrating the results of KEGG pathway enrichment analysis.

Following the standardization of all Andro target genes using the UniProt database, 443 genes were selected for further analysis. After merging and removing duplicates from the Genecards, CTD, and MalaCards databases, a total of 3238 chondrosarcoma-related genes were identified. The intersection of these two gene sets yielded 167 overlapping genes, which were considered potential Andro targets for chondrosarcoma treatment (**Figure 2A**).

### 3.2 Results of GO and KEGG analyses

GO enrichment analysis results were illustrated in **Figures 2B–D**, highlighting the top 10 biological functions associated with the potential targets of Andro. KEGG pathway enrichment analysis identified 169 signaling pathways implicated in Andro’s anti-cancer effects. Among these, the 15 most relevant pathways to chondrosarcoma were selected, including the PI3K/Akt, HIF-1, T-cell receptor, and PD-L1 expression/PD-1 checkpoint pathways (**Figure 2E**). These findings suggest that Andro exerts its therapeutic effects on chondrosarcoma through a broad array of biological functions and signaling pathways.

### 3.3 PPI network construction and core target analysis

**Figure 3:**
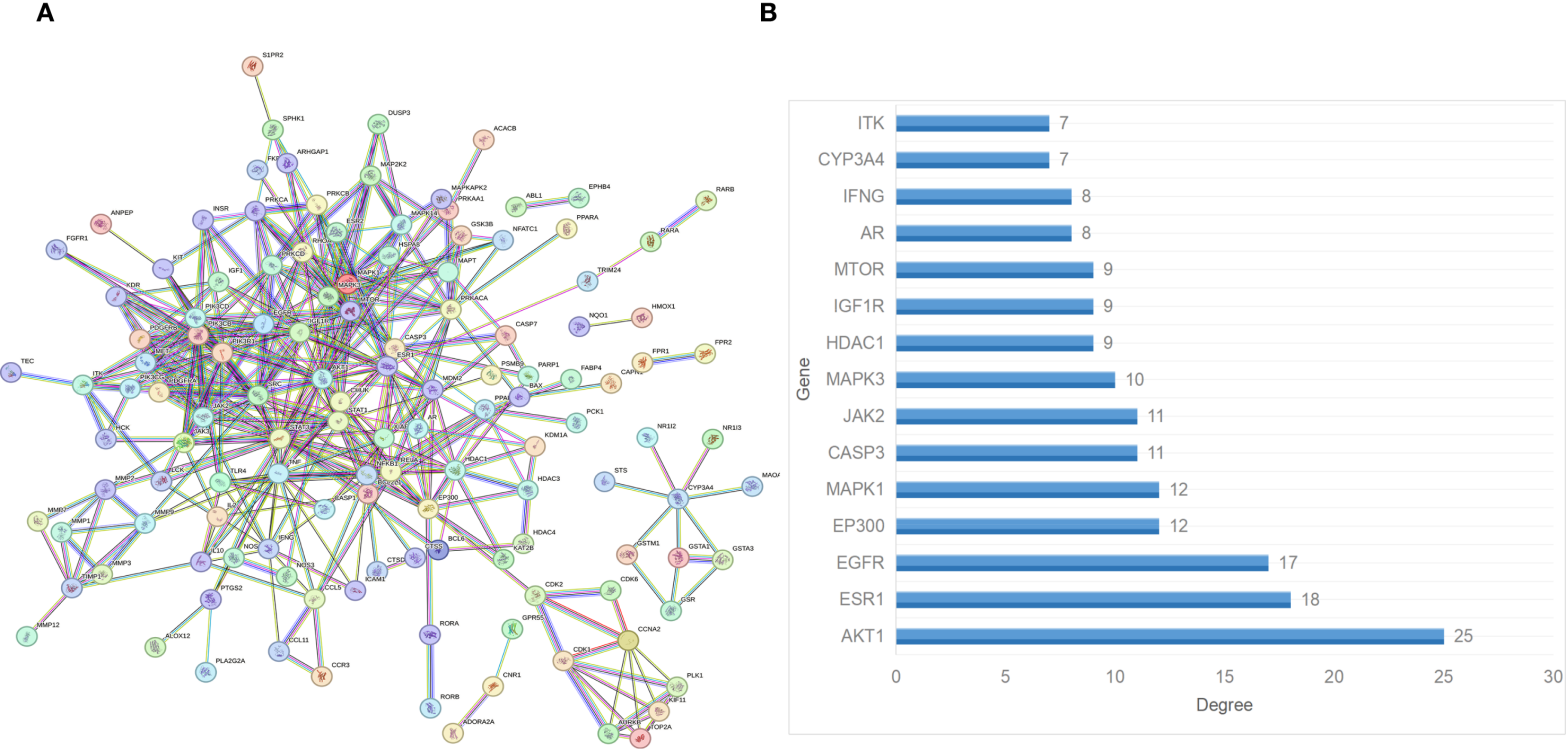
**(A)** PPI network of the overlapping target genes. **(B)** Histogram of the adjacent target number.

**Figure 4:**
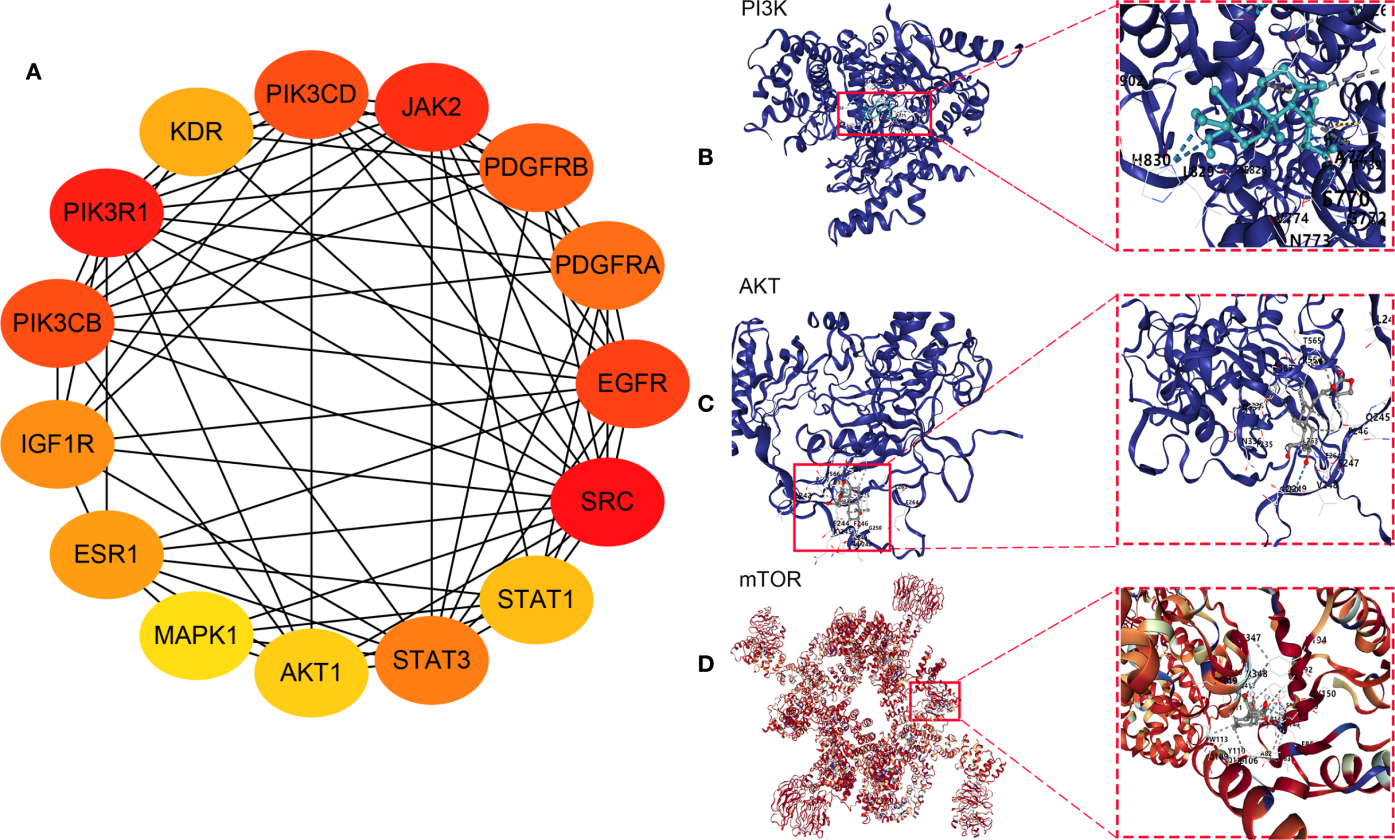
**(A)** Core genes identified using the cytoHubba plugin; Molecular docking between andrographolide and its core targets for the treatment of chondrosarcoma: **(B)** PI3K, **(C)** Akt, and **(D)** mTOR.

The potential targets of Andro for treating chondrosarcoma were imported into the STRING database to construct a PPI network. PPI analysis revealed an average degree of 4 for all targets. **Figure 3A** illustrated the PPI network, where all nodes were interconnected. The top 15 target genes identified were Akt, ESR1, EGFR, EP300, MAPK1, CASP3, JAK2, MAPK3, HDAC1, IGF1R, MTOR, AR, IFNG, CYP3A4, and ITK (**Figure 3B**). The PPI network was further visualized using Cytoscape software, and the top 15 core genes were determined through the cytoHubba plugin. These core genes included SRC, PIK3R1, JAK2, EGFR, PIK3CD, PIK3CB, PDGFRB, PDGFRA, STAT3, IGF1R, ESR1, KDR, STAT1, Akt, and MAPK1 (**Figure 4A**).

### 3.4 Results of molecular docking

Network pharmacology analysis identified PI3K, Akt, and mTOR as the primary therapeutic targets of Andro in chondrosarcoma for molecular docking. The docking results revealed that the binding energies of the Andro-PI3K, Andro-Akt, and Andro-mTOR complexes were<–5 kcal/mol (–7.4, –8.7, and –9.2, respectively), suggesting strong binding affinity between Andro and these core targets (**Figures 4B–D**).

### 3.5 Effects of Andro on chondrosarcoma cell migration and invasion

**Figure 5:**
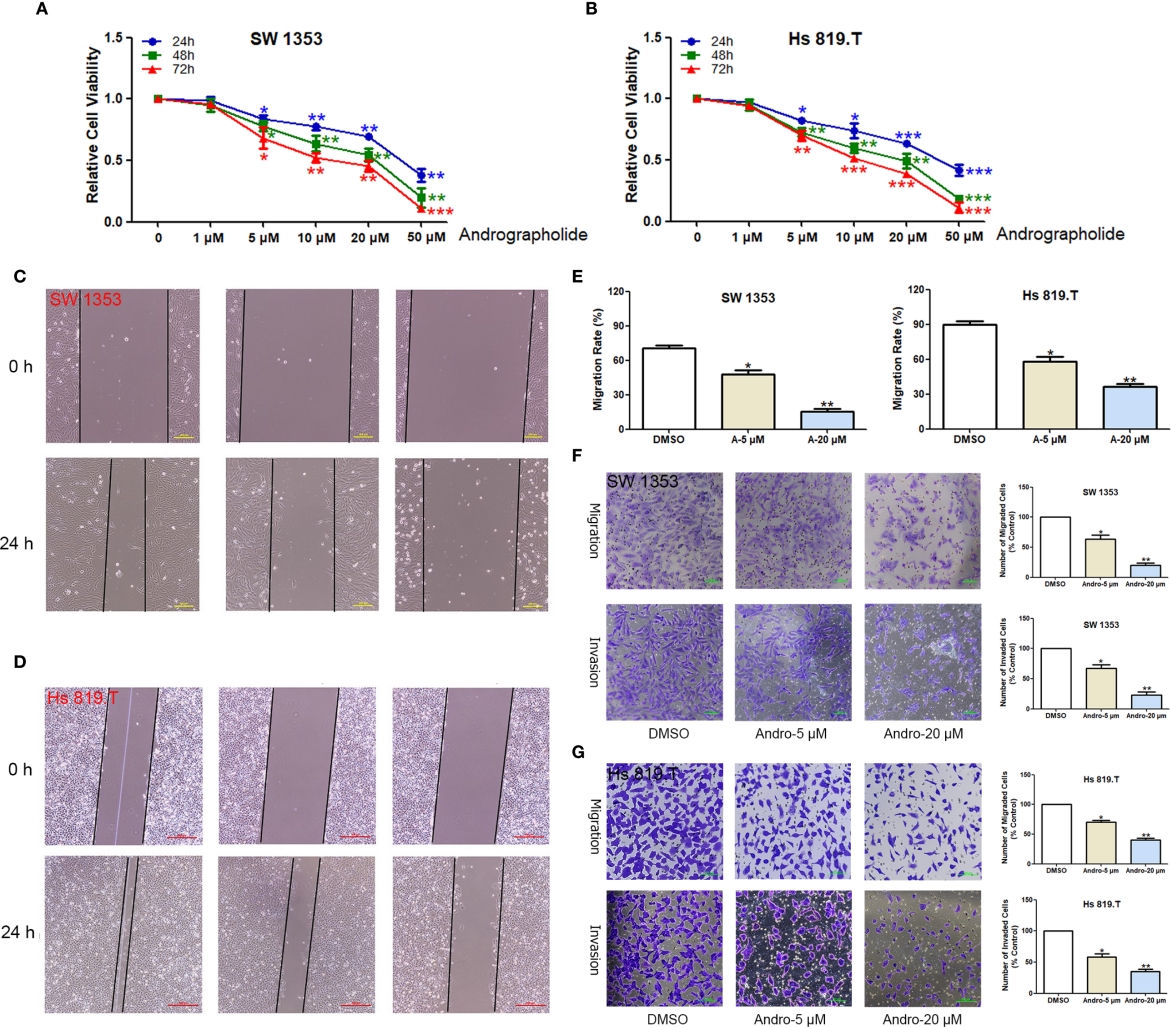
Andrographolide inhibited chondrosarcoma cell migration and invasion *in vitro*. The healing of scratches on cell monolayers was observed under an inverted microscope. **(A)** SW1353 and **(B)** Hs 819.T cells were treated with increasing concentrations (1, 5, 10, 20, and 50 μM) of Andro for the indicated times (24–72 h). The OD value was then determined by CCK-8 assay, and the relative inhibition rate was statistically analyzed. **(C)** SW1353 and **(D)** Hs 819.T cell monolayers were scratched and treated with different concentrations of Andro (5 and 20 μM) for 24 hours. **(E)** The wound healing rates of SW1353 and Hs 819.T cells were statistically analyzed. Each bar represents the mean ± SEM of three independent experiments. **(F)** Number of invasive cells. **(G)** Number of migrated cells. *p<0.05; **p<0.01 versus DMSO.

**Figure 6:**
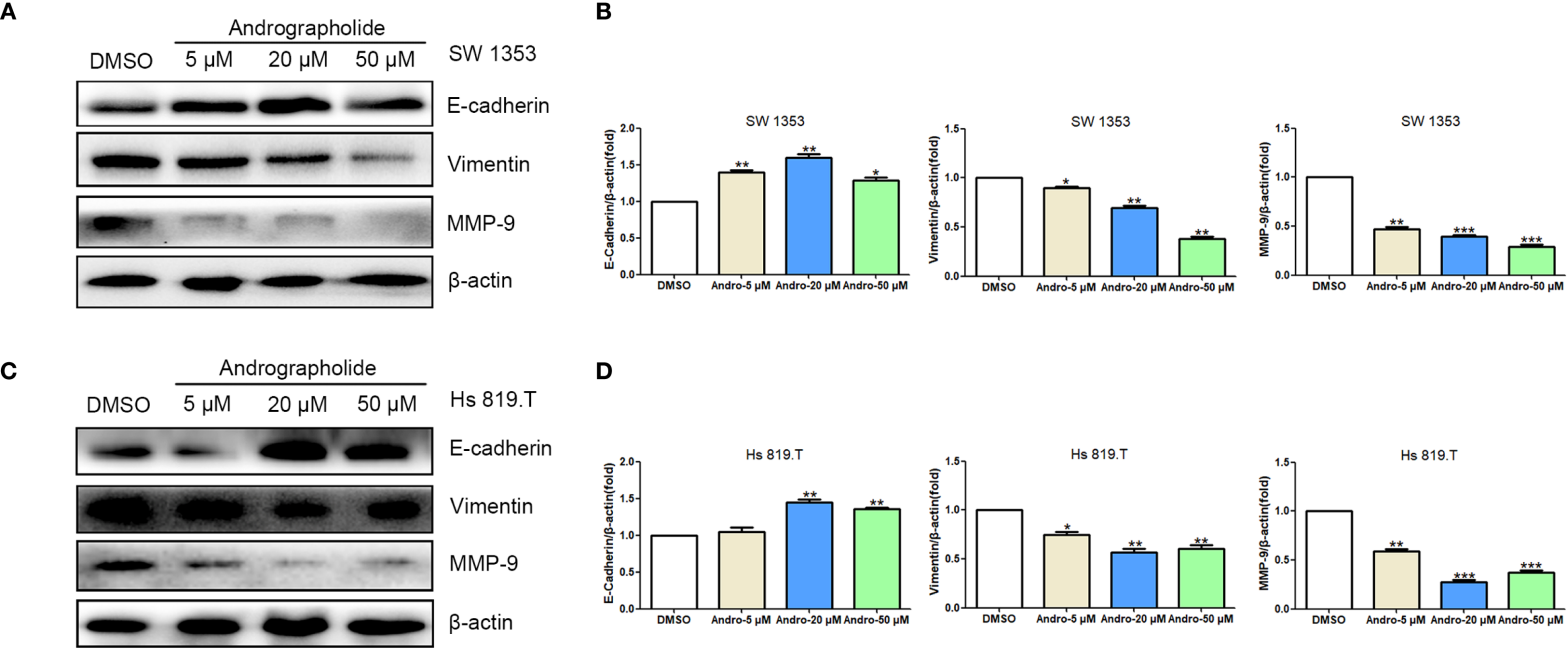
Andrographolide regulated the expression of cell migration-related proteins in chondrosarcoma. SW1353 **(A)** and Hs 819.T **(C)** cells were treated with different concentrations of Andro (5, 20, and 50 μM) for 24 h, and the expression of cell migration-related proteins (E-cadherin, vimentin, and MMP-9) was determined via western blotting (Representative images). **(B, D)** The expression of E-cadherin, vimentin, and MMP-9 in SW1353 **(B)** and Hs 819.T **(D)** cells was analyzed using the ImageJ software. Each bar represents the mean ± SEM of three independent experiments. Original blots are included in a **Supplementary Figures 1, 2**. *p<0.05; **p<0.01; ***p<0.001 versus DMSO.

CCK-8-based assays indicated that Andro at concentrations of 5, 10, 20, 50 μM significantly inhibited both SW1353 and Hs 819.T cells (**Figures 5A, B**). Wound healing assays revealed a reduction in the wound healing rates of SW1353 and Hs 819.T cells after 24 hours of treatment with 5- and 20-μM Andro (**Figures 5C–E**). Transwell migration and invasion assays demonstrated a marked decrease in the migratory and invasive capabilities of these cells following 24-hour treatment with 5- and 20-μM Andro. Notably, higher Andro concentrations correlated with a reduced number of migratory and invasive cells (**Figures 5F–G**). WB analysis indicated that treatment with 5-, 20-, and 50-μM Andro for 24 hours significantly downregulated the protein expression of vimentin and MMP-9, while upregulating E-cadherin expression in both chondrosarcoma cell lines (**Figure 6**).

### 3.6 Andro inhibited the mTOR/PI3K/Akt signaling pathway in chondrosarcoma cells

**Figure 7:**
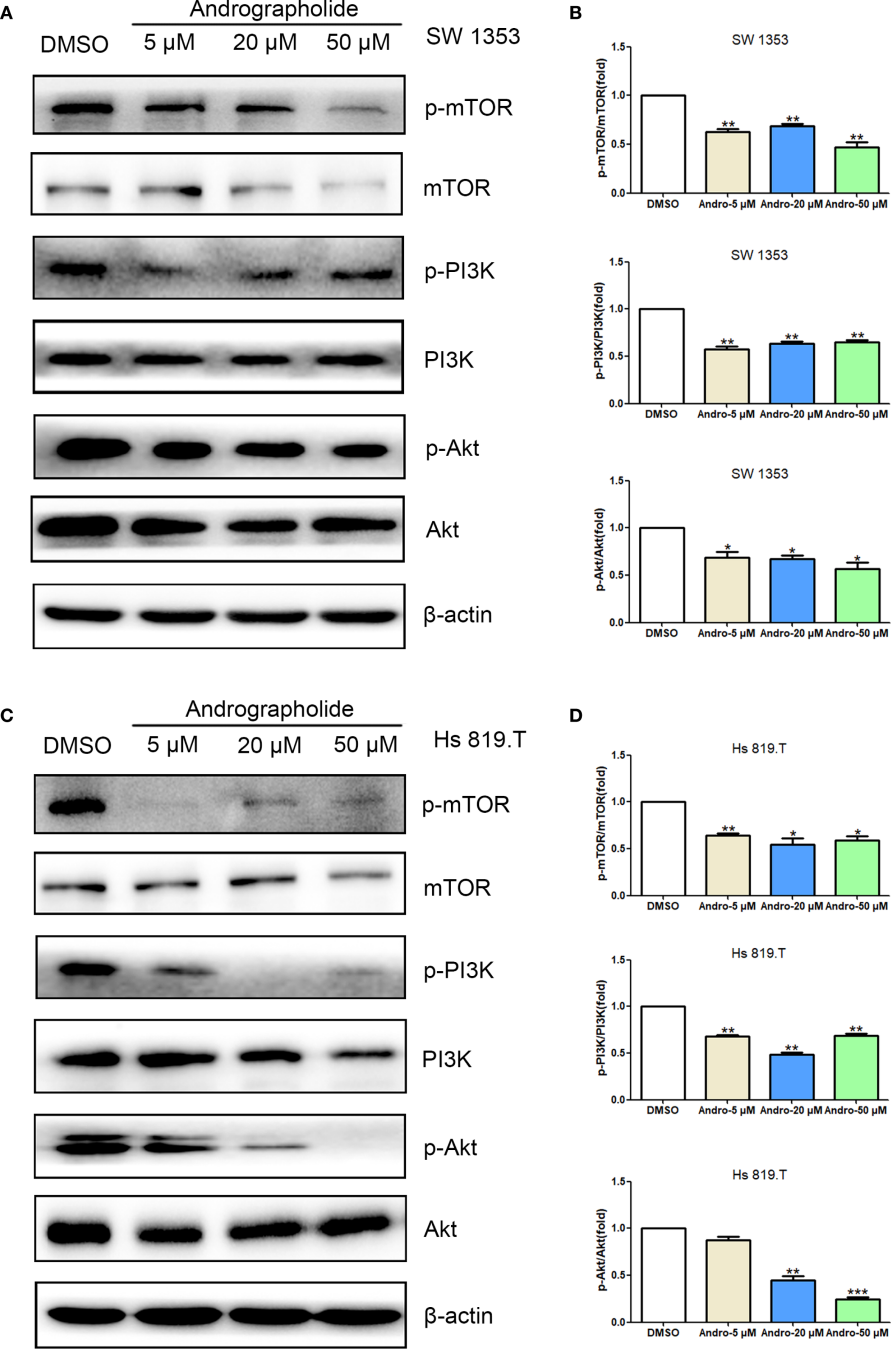
Andrographolide inhibited the mTOR/PI3K/Akt signaling pathway in chondrosarcoma. SW1353 **(A)** and Hs 819.T **(C)** cells were treated with different concentrations of Andro (5, 20, and 50 μM) for 24 h, and the protein and phosphorylation levels of mTOR, PI3K, and Akt were determined via western blotting (Representative images). **(B, D)** The p-mTOR/mTOR, p-PI3K/PI3K, and p-Akt/Akt ratios in SW1353 **(B)** and Hs 819.T **(D)** cells were analyzed using the ImageJ software. Each bar represents the mean ± SEM of three independent experiments. Original blots are included in a **Supplementary Figures 3, 4**. *p<0.05; **p<0.01; ***p<0.001 versus DMSO.

Network pharmacology analysis informed the investigation of Andro’s impact on the PI3K/Akt/mTOR signaling pathway in chondrosarcoma cells *in vitro*. WB analysis revealed that treatment with Andro for 24 hours at concentrations of 5, 20, and 50 μM reduced the p-mTOR/mTOR, p-PI3K/PI3K, and p-Akt/Akt ratios in both SW1353 and Hs 819.T cells (**Figure 7**). These results suggest that Andro suppresses chondrosarcoma cell migration and invasion by inhibiting the PI3K/Akt/mTOR signaling pathway.

### 3.7 Andro induces autophagy in chondrosarcoma cells

**Figure 8:**
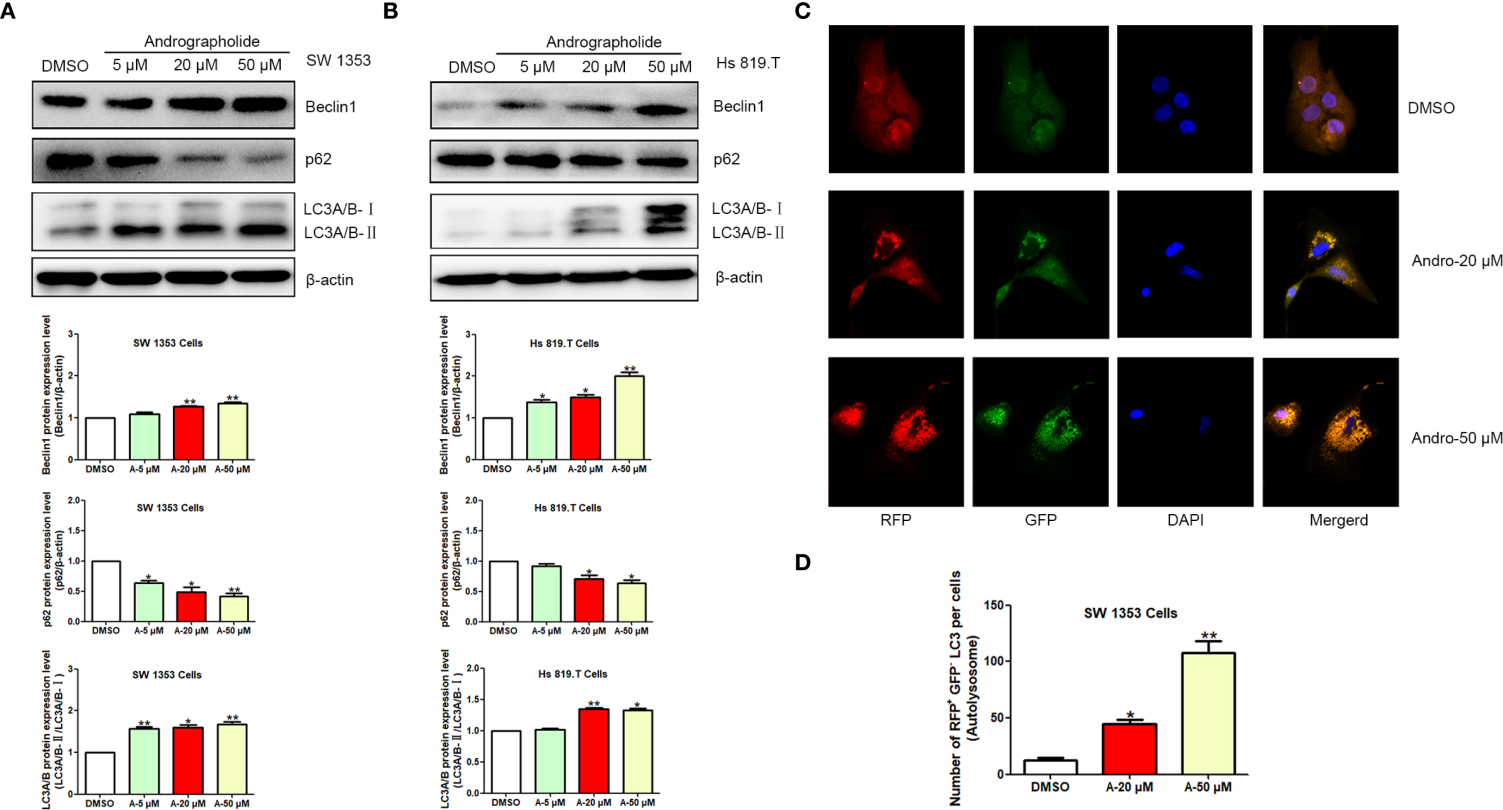
Andrographolide induces autophagy in chondrosarcoma cells. SW1353 **(A)** and Hs 819.T **(B)** cells were exposed to various concentrations of Andro (5, 20, and 50 μM) for 24 hours, and the protein expression levels of Beclin-1, p62, LC3A/B-II, and LC3A/B-I were assessed via Western blotting (Representative images). The Beclin-1 and p62 expression levels, along with the LC3A/B-II/LC3A/B-I ratios, were quantified using ImageJ software. Each bar represents the mean ± SEM from three independent experiments. Original blots are included in a **Supplementary Figures 5, 6**. **(C)** SW1353 cells were infected with mRFP-GFP-LC3 lentivirus and treated with Andro at concentrations of 20 and 50 μM for 24 hours. Cells were subsequently examined using confocal fluorescence microscopy (LSM710, Carl Zeiss, Germany). **(D)** Number of RFP^+^ GFP^-^ LC3 per cells (Autolysosome). Statistical significance is indicated as follows: *p<0.05; **p<0.01; ***p<0.001 versus DMSO.

Building on previous findings, it was hypothesized that Andro could inhibit chondrosarcoma cell migration and invasion, with Andro-induced autophagy serving as a key mechanism. To assess this, the effects of Andro on autophagy-related proteins in chondrosarcoma cells were examined *in vitro*. WB analysis revealed a dose-dependent decrease in p62 expression, while Beclin-1 levels and the LC3A/B-II/LC3A/B-I ratios increased following 24-hour treatment with Andro at concentrations of 5, 20, and 50 μM in both SW1353 and Hs 819.T cells (**Figures 8A, B**). Confocal microscopy further confirmed a marked increase in the red-to-green fluorescence ratio in Andro-treated cells, indicating enhanced autophagic flux (**Figures 8C, D**). These results suggest that Andro inhibits chondrosarcoma cell migration and invasion by downregulating the PI3K/Akt/mTOR pathway and promoting autophagy.

**Figure 9:**
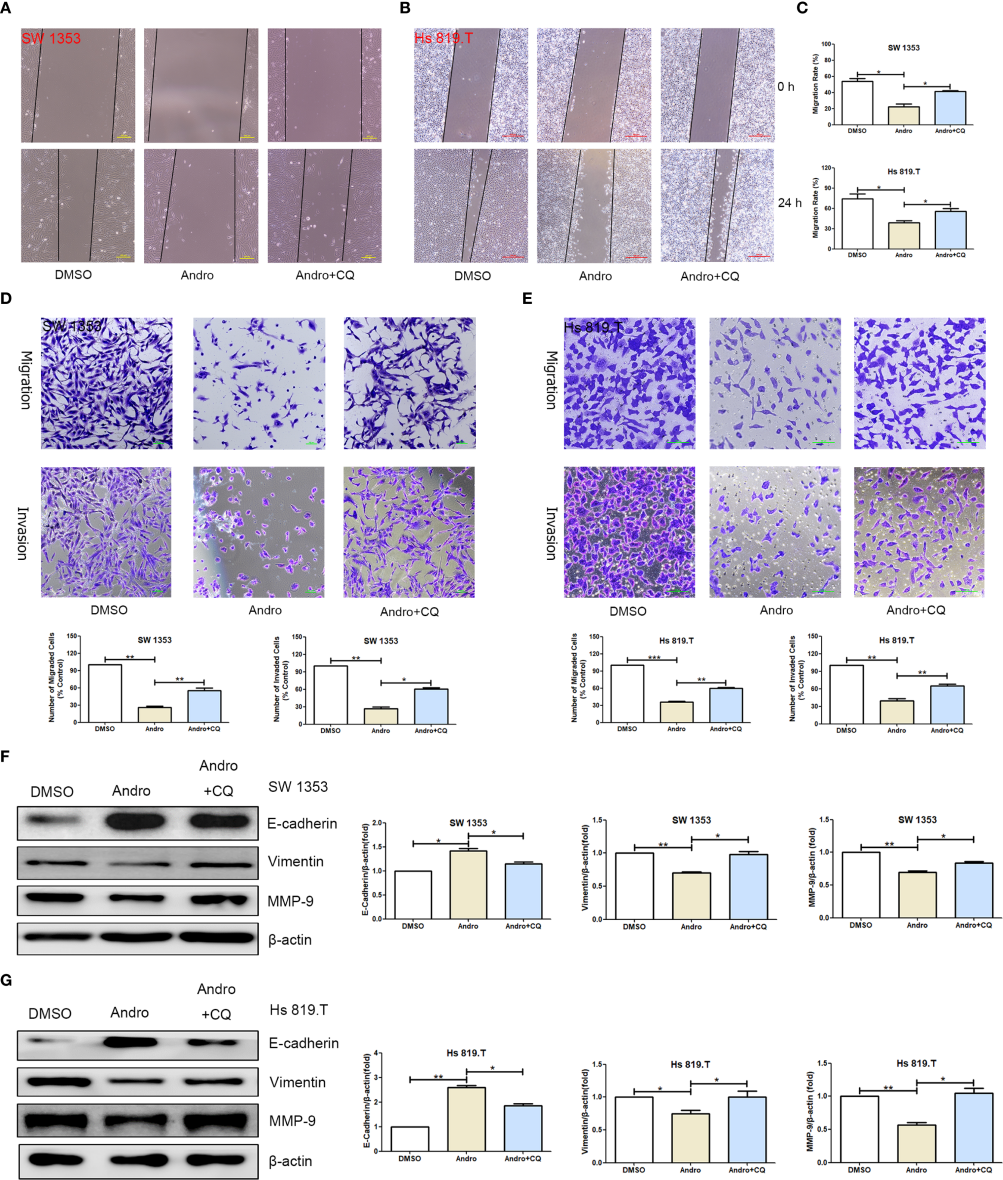
Inhibiting autophagy restores the migration and invasion abilities of chondrosarcoma cells that were suppressed by andrographolide. **(A)** SW1353 and **(B)** Hs 819.T cell monolayers were scratched and treated with Andro (20 μM) or chloroquine (CQ, 0.5 μM, MedChemExpress, HY-17589A) for 24 hours. **(C)** The wound healing rates of SW1353 and Hs 819.T cells were statistically analyzed. Each bar represents the mean ± SEM of three independent experiments. **(D)** Number of migrated and invasive SW1353 cells. **(E)** Number of migrated and invasive Hs 819.T cells. SW1353 **(F)** and Hs 819.T **(G)** cells were treated with Andro (20 μM) or CQ (0.5 μM) for 24 h, and the expression of cell migration-related proteins (E-cadherin, Vimentin, and MMP-9) was determined via western blotting (Representative images). The expression levels of E-cadherin, Vimentin, and MMP-9 were analyzed using the ImageJ software. Each bar represents the mean ± SEM of three independent experiments. Original blots are included in a **Supplementary Figures 8–11**. *p<0.05; **p<0.01; ***p<0.001 versus DMSO.

To establish a causal relationship between autophagy induction and the observed inhibition of migration, we incorporated autophagy inhibitor intervention into rescue experiments, with chloroquine (CQ, MedChemExpress, HY-17589A) serving as the autophagy inhibitor. To determine the optimal intervention concentration of CQ, we conducted cell viability assays. CCK-8 results indicated that the optimal concentration of CQ for intervening in SW1353 and Hs 819.T cells was 0.5 μM (**Supplementary Figure 7**). Scratch assay results showed that the effect of Andro was attenuated by the autophagy inhibitor CQ (**Figures 9A–C**). After CQ intervention, the number of migrated and invasive cells was higher than that in the Andro group (**Figures 9D, E**). WB results revealed that, on the basis of Andro treatment, CQ intervention significantly increased the expression of vimentin and MMP-9 proteins and downregulated the expression of E-cadherin protein in SW1353 and Hs 819.T cells (**Figures 9F, G**). Collectively, these results suggest that Andro exerts its pharmacological effects by inhibiting autophagy in chondrosarcoma cells.

## 4 Discussion

Surgical resection remains limited in the treatment of metastatic chondrosarcoma, often resulting in a poor prognosis (23, 24). Consequently, preventing invasion and metastasis across all pathological stages of chondrosarcoma holds significant clinical relevance. Although Andro exhibits potential anti-cancer properties, its pharmacological effects in chondrosarcoma remain inadequately explored. Previous research has demonstrated that Andro inhibits chondrosarcoma cell proliferation and promotes apoptosis (18). Building upon this evidence, the current study investigates the impact of Andro on chondrosarcoma cell migration and invasion. The findings indicate that Andro diminishes the migratory and invasive capabilities of chondrosarcoma cells by inhibiting the PI3K/Akt/mTOR signaling pathway. These results may contribute to advancing Andro as a therapeutic agent for chondrosarcoma treatment.

Network pharmacology identified PI3K, Akt, and mTOR as central targets of Andro in chondrosarcoma treatment. Numerous studies have established the significant roles of PI3K, Akt, and mTOR in chondrosarcoma metastasis (25–27). Qu et al. (25) demonstrated that PRKCI was overexpressed in osteosarcoma cell lines, and its silencing suppressed the proliferation, migration, and invasion of osteosarcoma cells. Moreover, they identified that PRKCI interacted with SQSTM1, modulating osteosarcoma cell proliferation via the Akt/mTOR signaling pathway. Truong et al. (26) emphasized that the IGF/PI3K/mTOR pathways were crucial in chondrosarcoma, with activation of the PI3K/Akt/mTOR pathway correlating with invasiveness and drug resistance. Additionally, interactions between PRKCI and the Akt/mTOR pathway in osteosarcoma were confirmed (27), highlighting the relevance of this pathway in both osteosarcoma and chondrosarcoma. Given that osteosarcoma and chondrosarcoma are both primary malignant bone tumors (27), they share common biological traits and signaling pathway activations. Collectively, these studies reinforce the pivotal roles of PI3K, Akt, and mTOR in chondrosarcoma metastasis. KEGG pathway enrichment analysis further indicated that the PI3K-Akt signaling pathway was a primary pathway linked to the core target genes. The PI3K/Akt/mTOR pathway is a well-known signaling cascade regulating cell proliferation and apoptosis, playing a critical role in the initiation and progression of various cancers (28, 29). Zhang et al. demonstrated that upon activation, PI3K activated Akt, triggering its translocation to the cell membrane, where it was subsequently activated and underwent auto-phosphorylation (30). Akt thus functions as a key downstream kinase of PI3K (30). Activation of the PI3K/Akt signaling pathway induces the upregulation of intracellular p-Akt (31, 32), which contributes to cell proliferation, apoptosis, transcription, translation, the cell cycle, and vascular growth across various cancers. Moreover, mTOR signaling pathway activation is implicated in the development of multiple malignancies. mTOR is typically upregulated in tumors and suppresses autophagy. Kopecky et al. observed higher mTOR expression in kidney cancer tissues compared to normal kidney tissues (33) and confirmed mTOR as a potential drug target for renal cancer treatment. The PI3K/Akt pathway, a classical apoptotic signaling route, is upstream of mTOR and plays a critical role in its activation (34). The PI3K/Akt/mTOR signaling pathway regulates the initiation and modulation of autophagy; thus, inhibiting this pathway can enhance cellular autophagy (34, 35). Zhu et al. demonstrated that baicalin inhibited the PI3K/Akt/mTOR pathway, inducing apoptosis in human chondrosarcoma cells (36). Similarly, Wu et al. reported that endothelin-1 regulated the FAK/PI3K/Akt/mTOR pathway, promoting MMP-13 expression and migration in human chondrosarcoma cells (37). Furthermore, Kouvaras et al. identified the HIF-1 signaling pathway as a key regulator of chondrosarcoma angiogenesis and malignant progression through VEGF expression, highlighting its potential as a therapeutic target (38). The T-cell receptor signaling pathway also modulates the immune microenvironment and response to chondrosarcoma treatment, suggesting new avenues for immunotherapy (39). These pathways present valuable research prospects for chondrosarcoma treatment (38, 39). Based on network pharmacology findings, drugs targeting the PI3K/Akt/mTOR pathway represent promising therapeutic agents for chondrosarcoma.

Proliferation is a defining feature of tumor cells and a key factor in tumor malignancy. In this study, wound healing and transwell assays demonstrated that Andro significantly reduced the migration and invasion of chondrosarcoma cells *in vitro*. WB analysis revealed that Andro downregulated vimentin and MMP-9, while upregulating E-cadherin in chondrosarcoma cells. Autophagy inhibitor CQ effectively reversed the inhibitory effects of Andro on cell migration and invasion, highlighting that Andro acts through autophagy modulation. These findings underscore the critical role of autophagy in mediating Andro’s anticancer effects, suggesting that autophagy inhibition is a key mechanism by which Andro suppresses chondrosarcoma progression. Additionally, Andro decreased the p-mTOR/mTOR, p-PI3K/PI3K, and p-Akt/Akt ratios in these cells. Collectively, these results suggest that Andro suppresses the migratory and invasive capabilities of chondrosarcoma cells by inhibiting the PI3K/Akt/mTOR signaling pathway. These results align with those of Zhu et al., who demonstrated that inhibition of the PI3K/Akt/mTOR pathway reduced the metastatic and invasive potential of chondrosarcoma cells (36). Moreover, Zheng et al. reported that curcumin induced autophagy through the inhibition of the PI3K/Akt/mTOR pathway in gastric cancer (40). Moreover, Andro’s dual action of inducing autophagy and inhibiting the PI3K/Akt/mTOR pathway may effectively overcome resistance mechanisms in chondrosarcoma, offering a more comprehensive therapeutic effect than single-target inhibitors.

In this study, *in vitro* experiments demonstrated that Andro significantly induced autophagy in chondrosarcoma cells, providing novel insights into its anti-chondrosarcoma mechanisms. Autophagy, a process of cellular self-degradation, preserves homeostasis by degrading damaged organelles and protein aggregates (41). The expression of autophagy-related proteins p62, Beclin-1, and LC3A/B was assessed through WB. Results revealed that Andro treatment notably reduced p62 expression while increasing Beclin-1 levels and the LC3A/B-II to LC3A/B-I ratio. Given that p62 functions as a selective autophagy receptor (42), its reduction suggests enhanced autophagic degradation. Beclin-1, a critical initiator of autophagy, shows increased expression during autophagy activation (43). An elevated LC3A/B-II to LC3A/B-I ratio reflects increased autophagosome formation. Furthermore, Andro treatment significantly boosted autophagic flux, as evidenced by a marked increase in the red/green fluorescence ratio, indicating that Andro promotes both autophagosome formation and substrate degradation. Previous studies have also suggested that promoting autophagy can hinder the pathological progression of chondrosarcoma (44, 45). Sun et al. (44) reported that GANT-61 inhibited chondrosarcoma growth by inducing autophagy through suppression of the RNAP III pathway and tRNA-Gly-CCC synthesis, involving ULK1 and MAPK pathways. Pang et al. (45) demonstrated that vitamin E subtypes (AnTT, γ-T3, and δ-T3) triggered autophagy in chondrosarcoma cells by activating pathways such as ER stress and the unfolded protein response, thereby inhibiting cell proliferation and tumor development. As a key regulator of autophagy, the PI3K/Akt/mTOR pathway generally suppresses autophagy when activated (46, 47), whereas its inhibition promotes autophagy. Our results demonstrate that Andro significantly induces autophagy in chondrosarcoma cells, which is associated with the inhibition of cell migration and invasion. However, it should be noted that autophagy is a context-dependent process. While autophagy can promote tumor survival under certain stress conditions, as reported in some studies, our findings suggest that Andro-induced autophagy exerts a tumor-suppressive effect in chondrosarcoma cells. This discrepancy may be attributed to the specific signaling pathways modulated by Andro. In particular, Andro inhibits the PI3K/Akt/mTOR signaling pathway, which is known to suppress autophagy when activated. By downregulating this pathway, Andro promotes autophagy, leading to the degradation of pro-metastatic proteins such as vimentin and MMP-9, and the upregulation of E-cadherin, thereby inhibiting the metastatic potential of chondrosarcoma cells. Therefore, the autophagy induced by Andro appears to be beneficial in the context of chondrosarcoma treatment, highlighting the importance of understanding the context-dependent outcomes of autophagy in different tumor settings. Our findings suggest that Andro’s inhibition of the PI3K/Akt/mTOR pathway may offer a potential strategy to address metastasis in chondrosarcoma, a tumor type resistant to conventional therapies. These results provide a rational basis for exploring Andro’s clinical relevance in improving patient outcomes.

This study presents several limitations that warrant consideration. Firstly, our study’s findings are currently limited to *in vitro* experiments, and the link between Andro-induced autophagy and migration inhibition lacks direct *in vivo* validation. Future work will include autophagy inhibitor experiments and knockout models to directly demonstrate this link, as well as *in vivo* studies using genetic or chondrosarcoma-specific mouse models to further elucidate Andro’s mechanisms. Secondly, the databases employed for network pharmacology are continually updated, implying that the bioinformatics analysis is based on the current data available. Thirdly, the KEGG pathway enrichment analysis identifies multiple pathways (such as the HIF-1 and FoxO signaling pathways) beyond the PI3K/Akt signaling pathway, necessitating further validation of these pathways as a key area for future research. Finally, the use of inhibitors or activators to target core signaling pathways, followed by a comparison of their effects with those of Andro, could clarify Andro’s mechanism and efficacy. This approach is essential for advancing future research, particularly *in vitro* studies.

## 5 Conclusion

This study demonstrates that Andro can inhibit chondrosarcoma cell migration and invasion, likely through the suppression of the PI3K/Akt/mTOR pathway, alleviation of autophagy inhibition, and activation of autophagy. This discovery provides a novel molecular mechanism underlying Andro’s anti-chondrosarcoma effects and supports the development of autophagy-targeted cancer therapies. Future research should evaluate Andro’s *in vivo* antitumor efficacy and explore its potential synergistic effects with other anticancer agents. Further investigation into Andro’s long-term influence on autophagy and its role within the tumor microenvironment will contribute to a deeper understanding of its clinical application potential.

## Data Availability

The original contributions presented in the study are included in the article/**Supplementary Material**. Further inquiries can be directed to the corresponding author.
