# Supplementary material for: Andrographolide suppresses chondrosarcoma cell migration and invasion by inhibiting the PI3K/Akt/mTOR signaling pathway and activating autophagy
Source: Front Oncol. 2025 Sep 26;15:1622666. doi: 10.3389/fonc.2025.1622666 (PMC12511062; doi:10.3389/fonc.2025.1622666)

Supplementary Figure 1
SW 1353

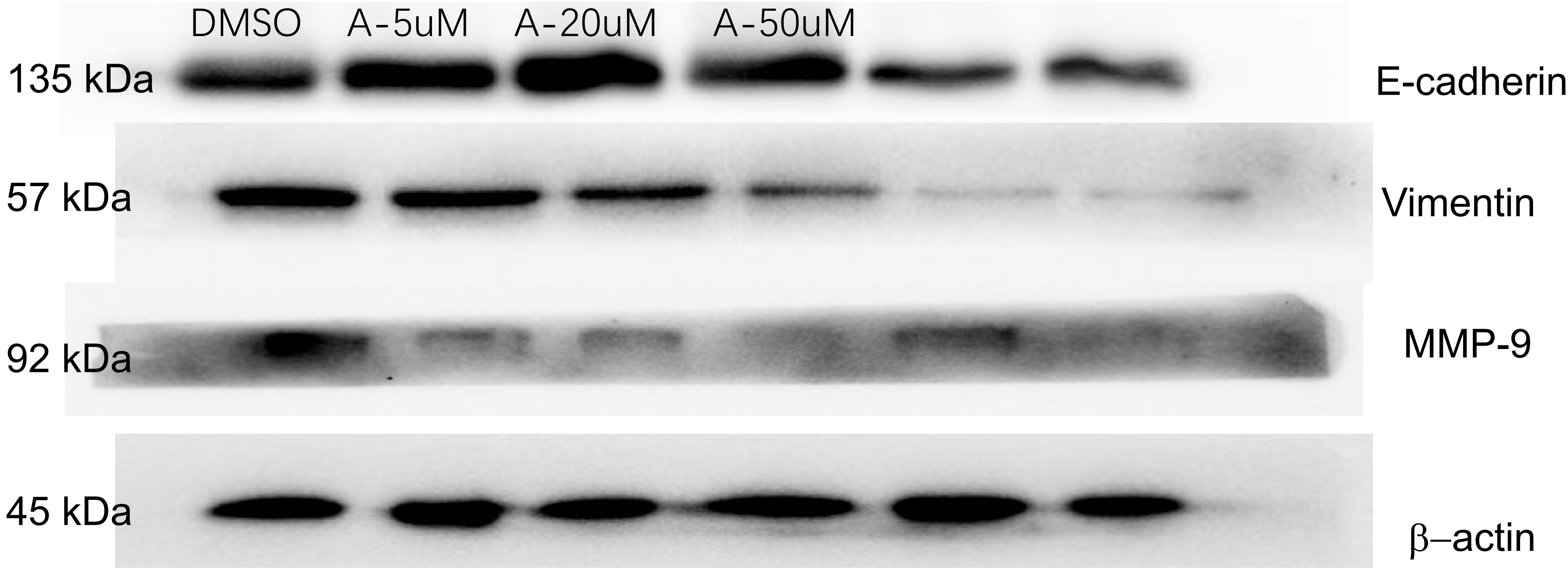

Supplementary Figure 2
HS819.T

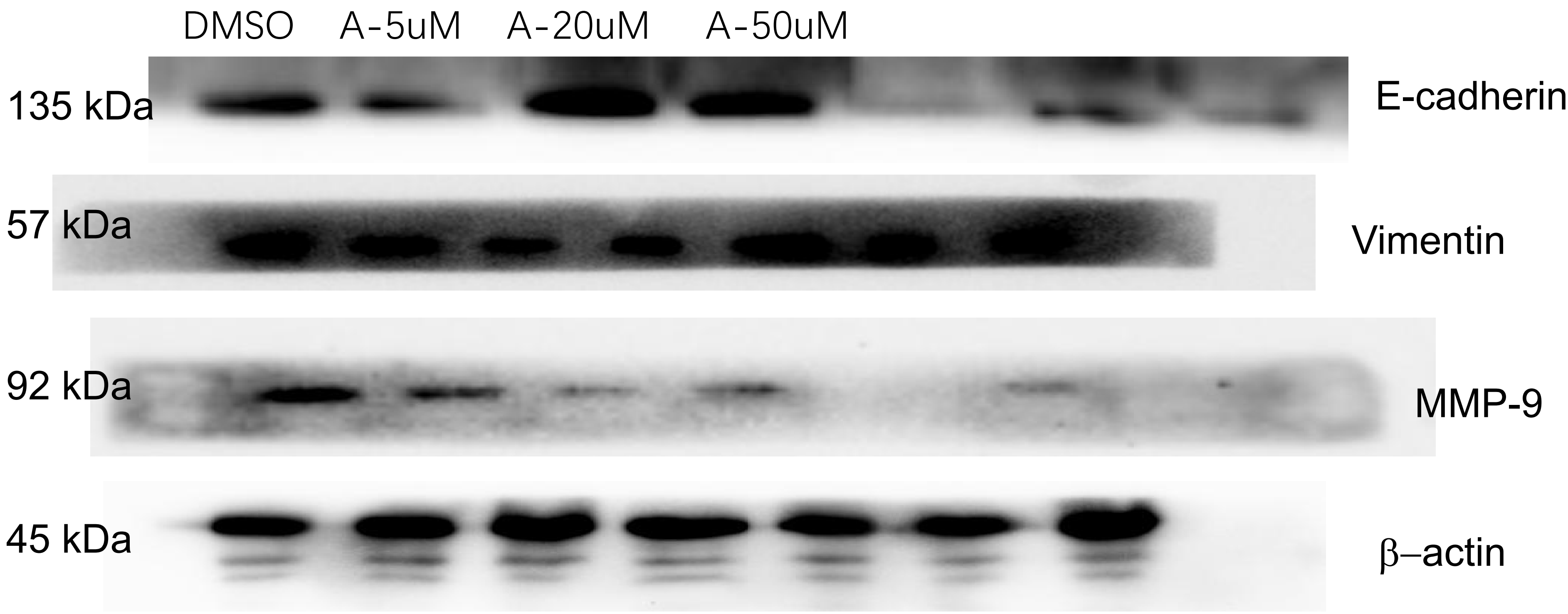

Supplementary Figure 3
SW 1353

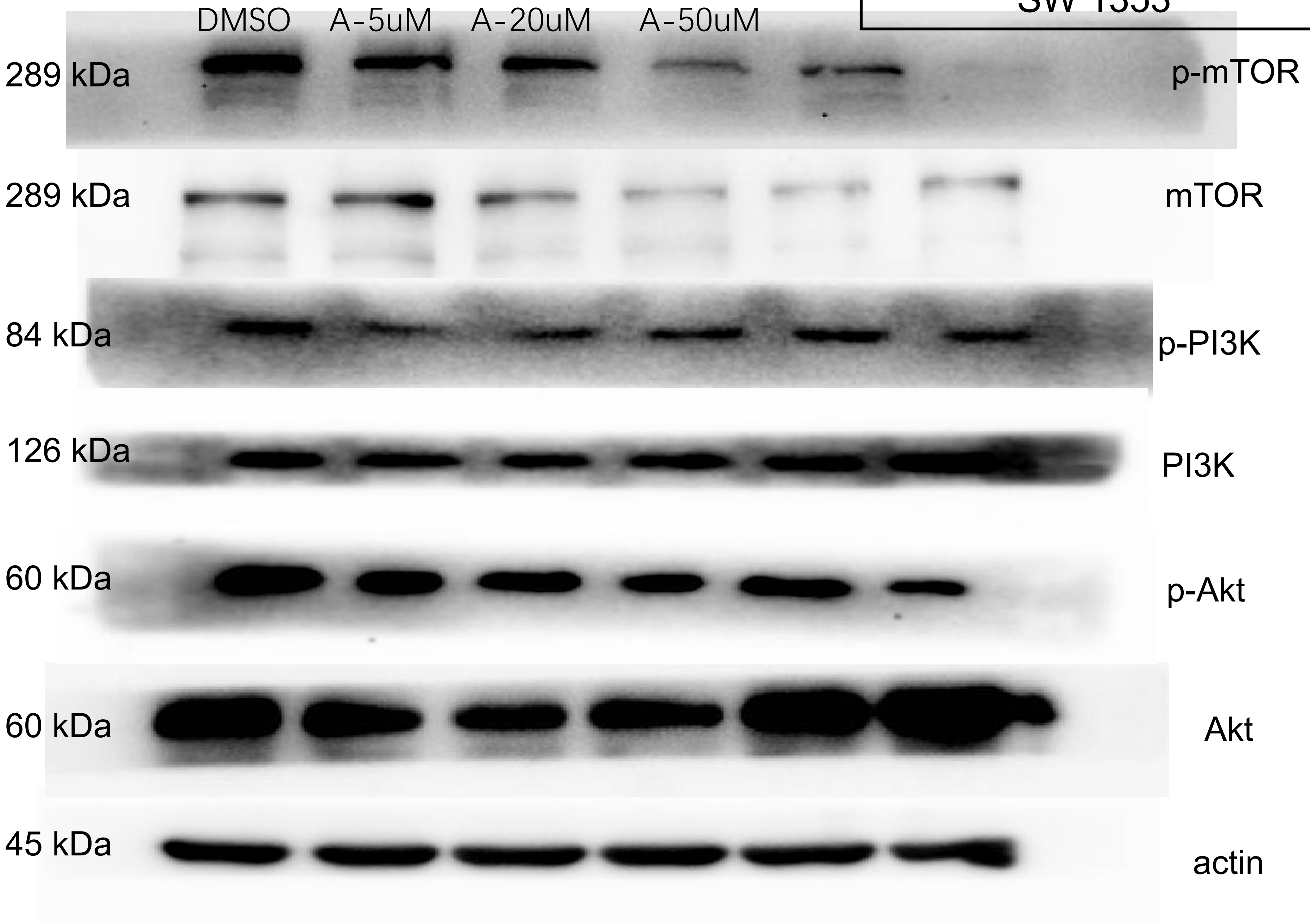

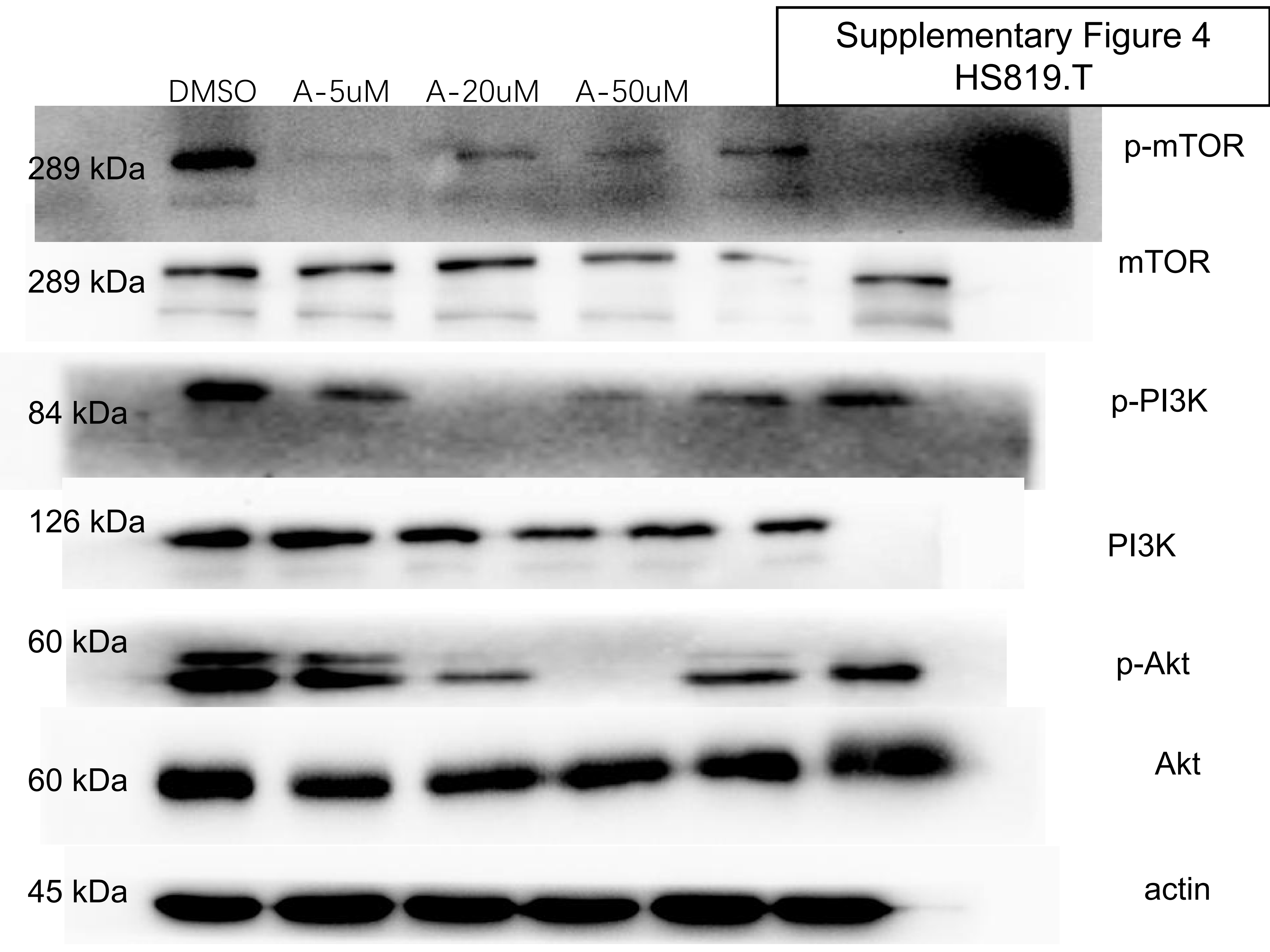
Supplementary Figure 4
HS819.T
DMSO
A-5uM
A-20uM
A-50uM
289 kDa
p-mTOR
289 kDa
mTOR
84 kDa
p-PI3K
126 kDa
PI3K
60 kDa
p-Akt
60 kDa
Akt
45 kDa
actin

Supplementary Figure 5
SW 1353

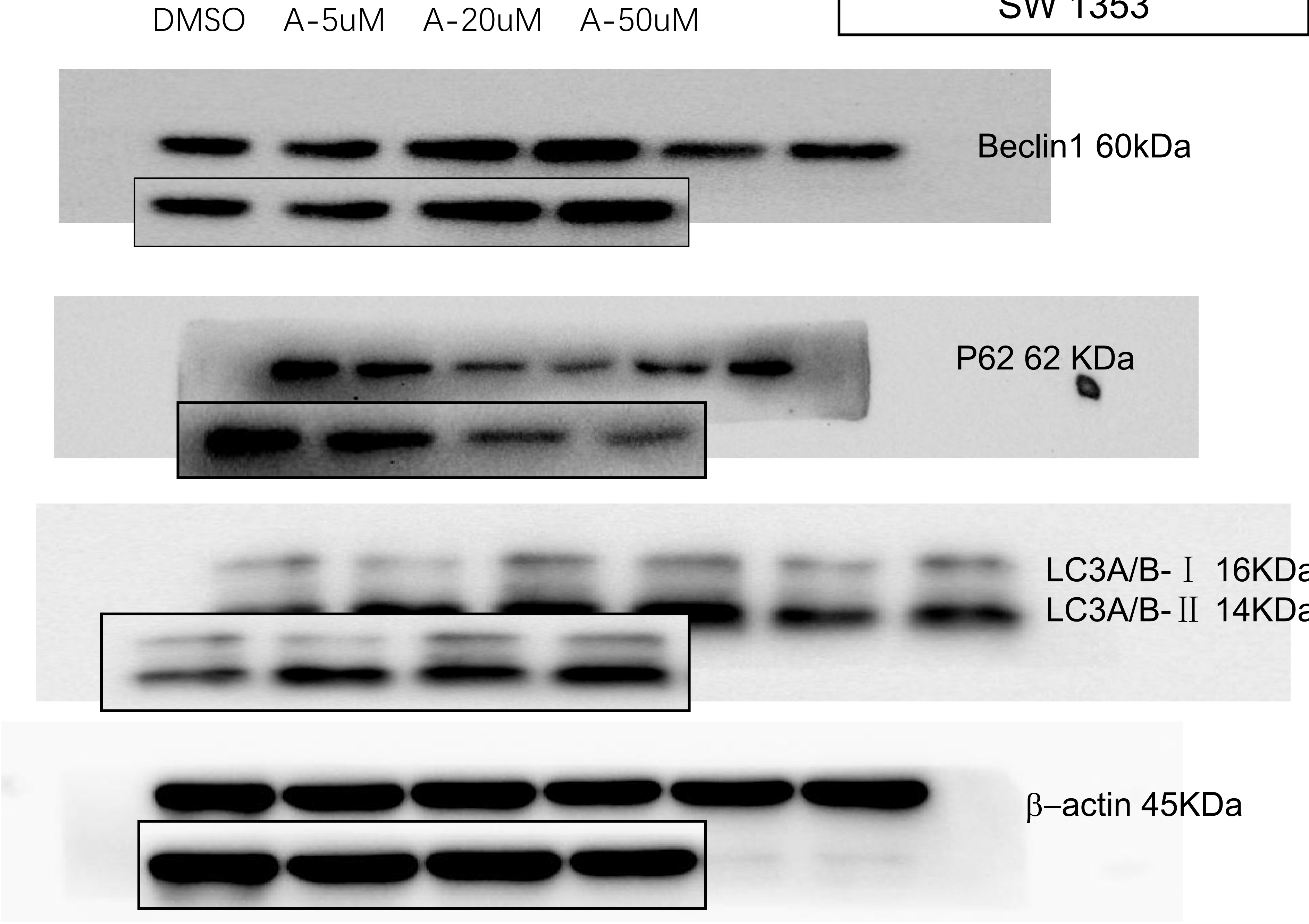

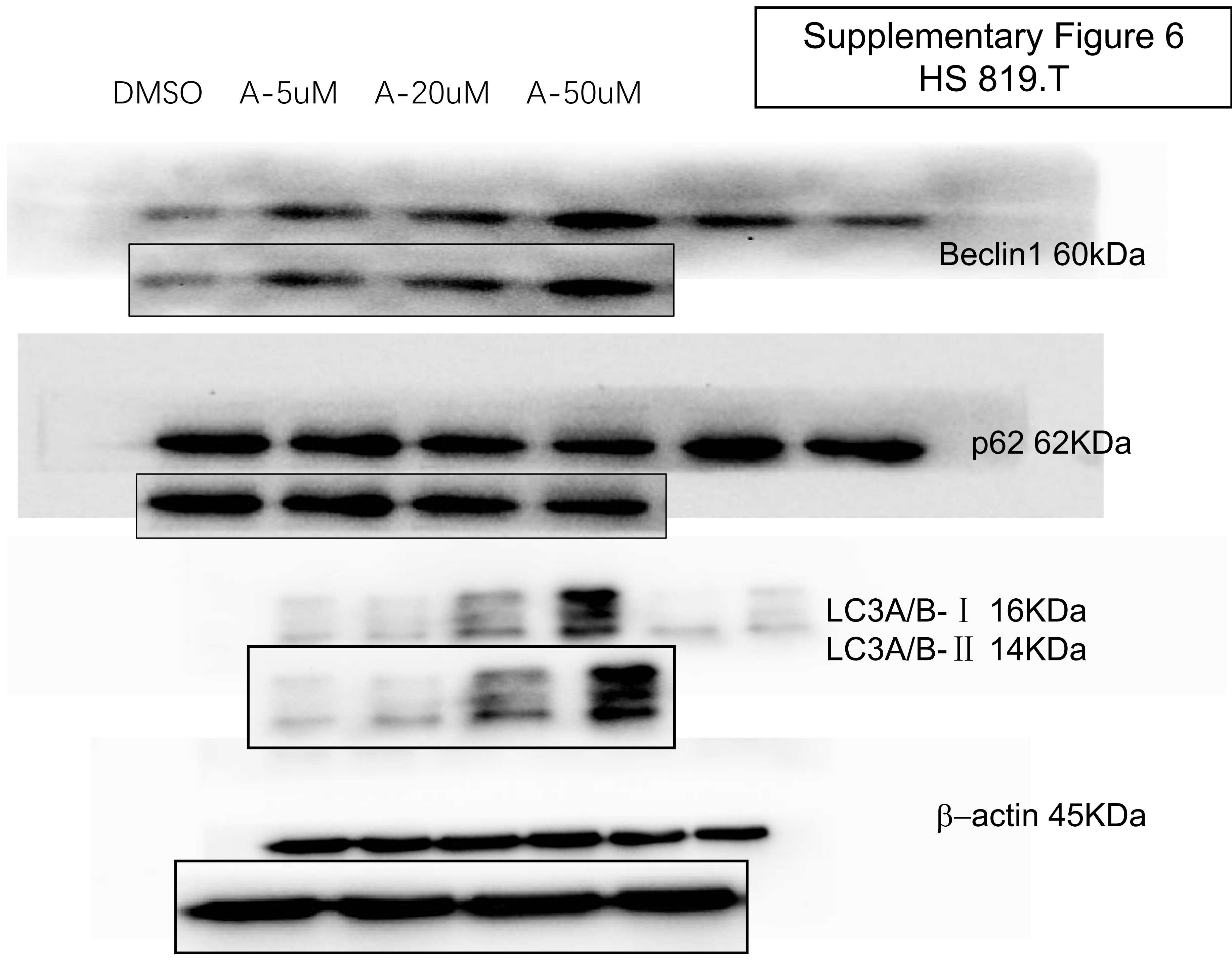
Supplementary Figure 6
HS 819.T
DMSO
A-5uM
A-20uM
A-50uM
Beclin1 60kDa
p62 62KDa
LC3A/B-Ⅰ 16KDa
LC3A/B-Ⅱ 14KDa
β–actin 45KDa

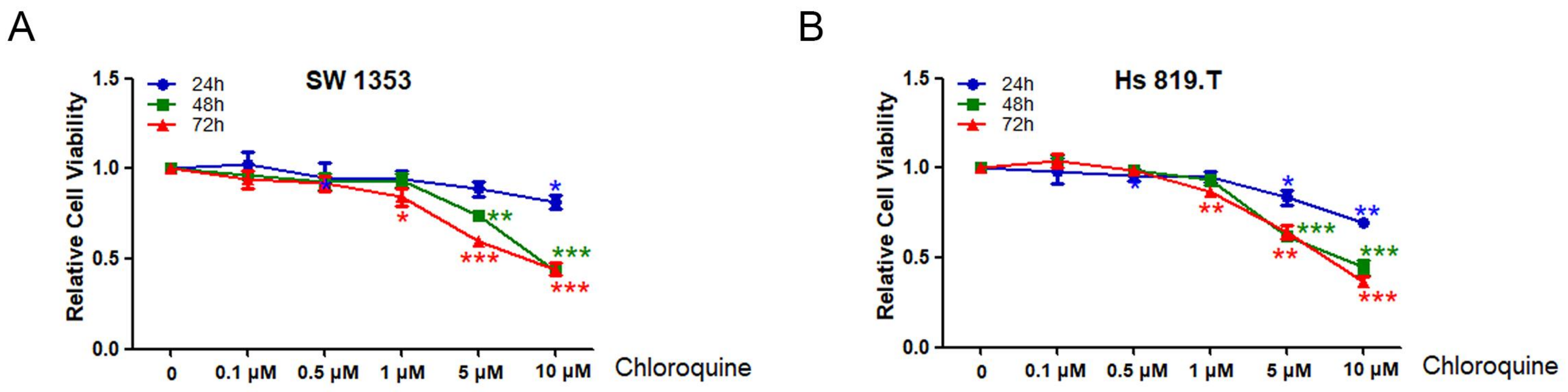

Supplementary Figure 7 (A) SW1353 and (B) Hs 819.T cells were treated with increasing concentrations (0.1, 0.5, 1, 5, and 10 μM) of Chloroquine (CQ) for the indicated times (24–72 h). The OD value was then determined by CCK-8 assay, and the relative inhibition rate was statistically analyzed. Each bar represents the mean ± SEM of three independent experiments. Note: *, $p < 0.05$; **, $p < 0.01$; ***, $p < 0.001$ versus DMSO.

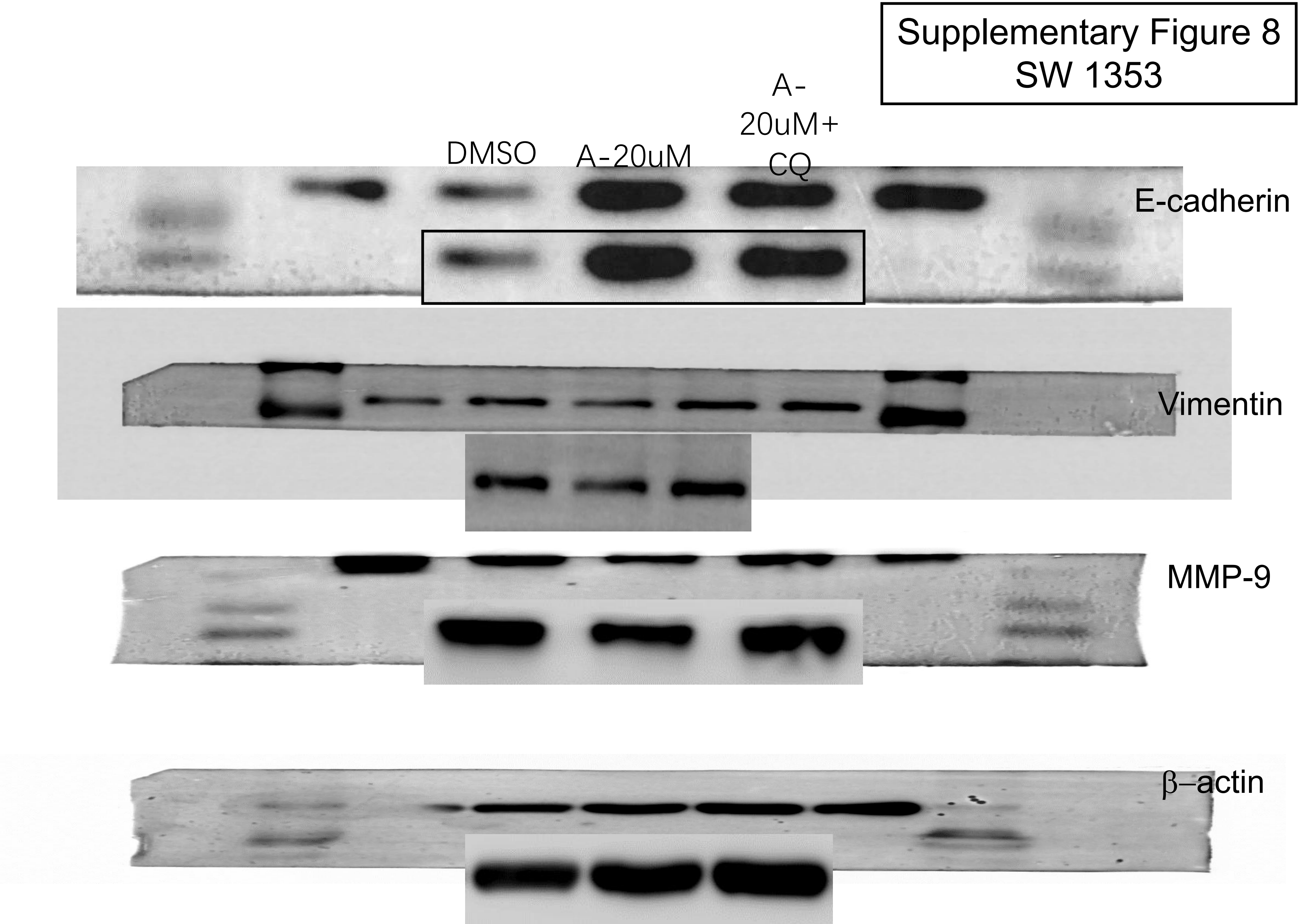
Supplementary Figure 8
SW 1353
DMSO
A-20uM
A-
20uM+
CQ
E-cadherin
Vimentin
MMP-9
β–actin

Supplementary Figure 9
SW 1353

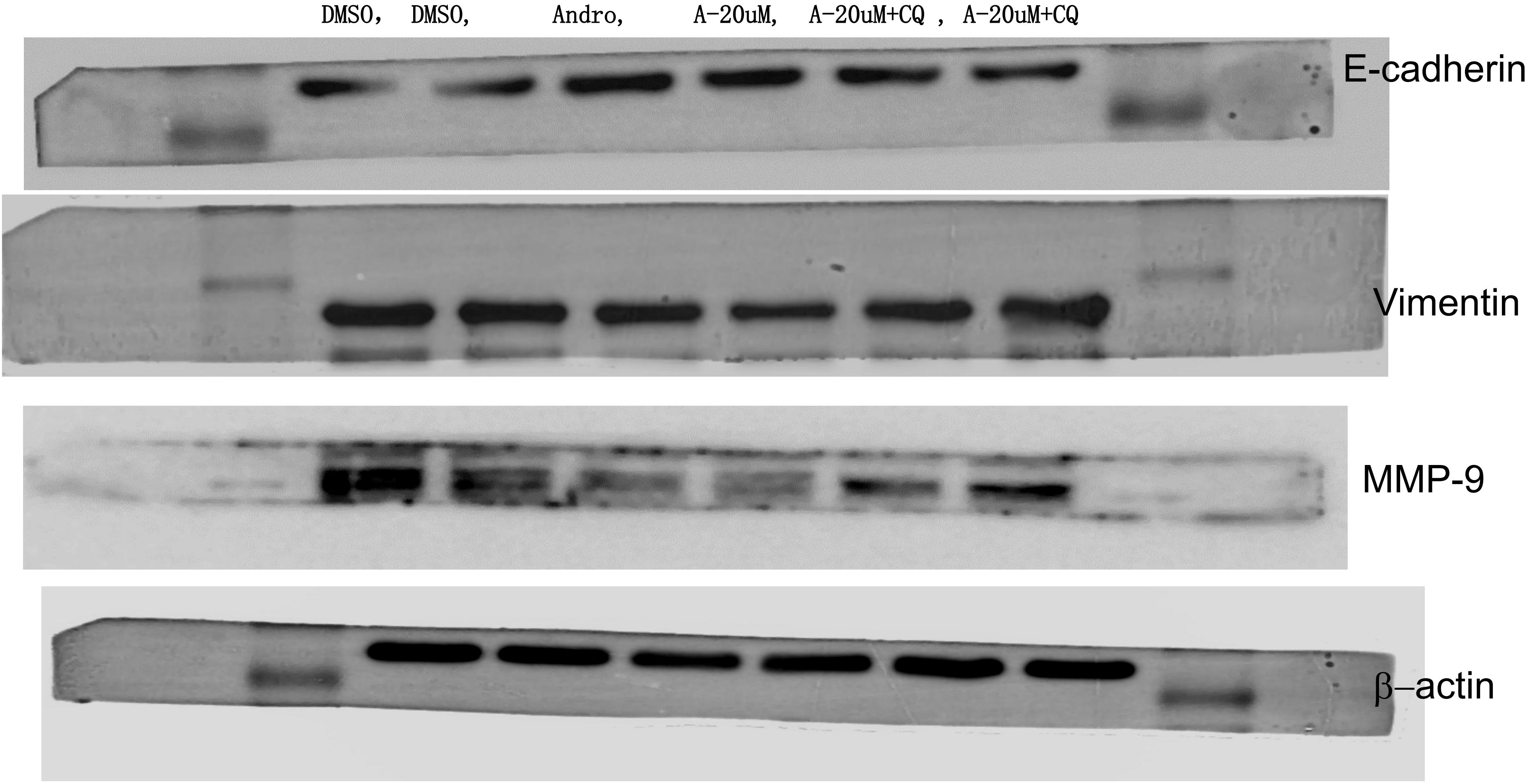

Supplementary Figure 10 HS819.T

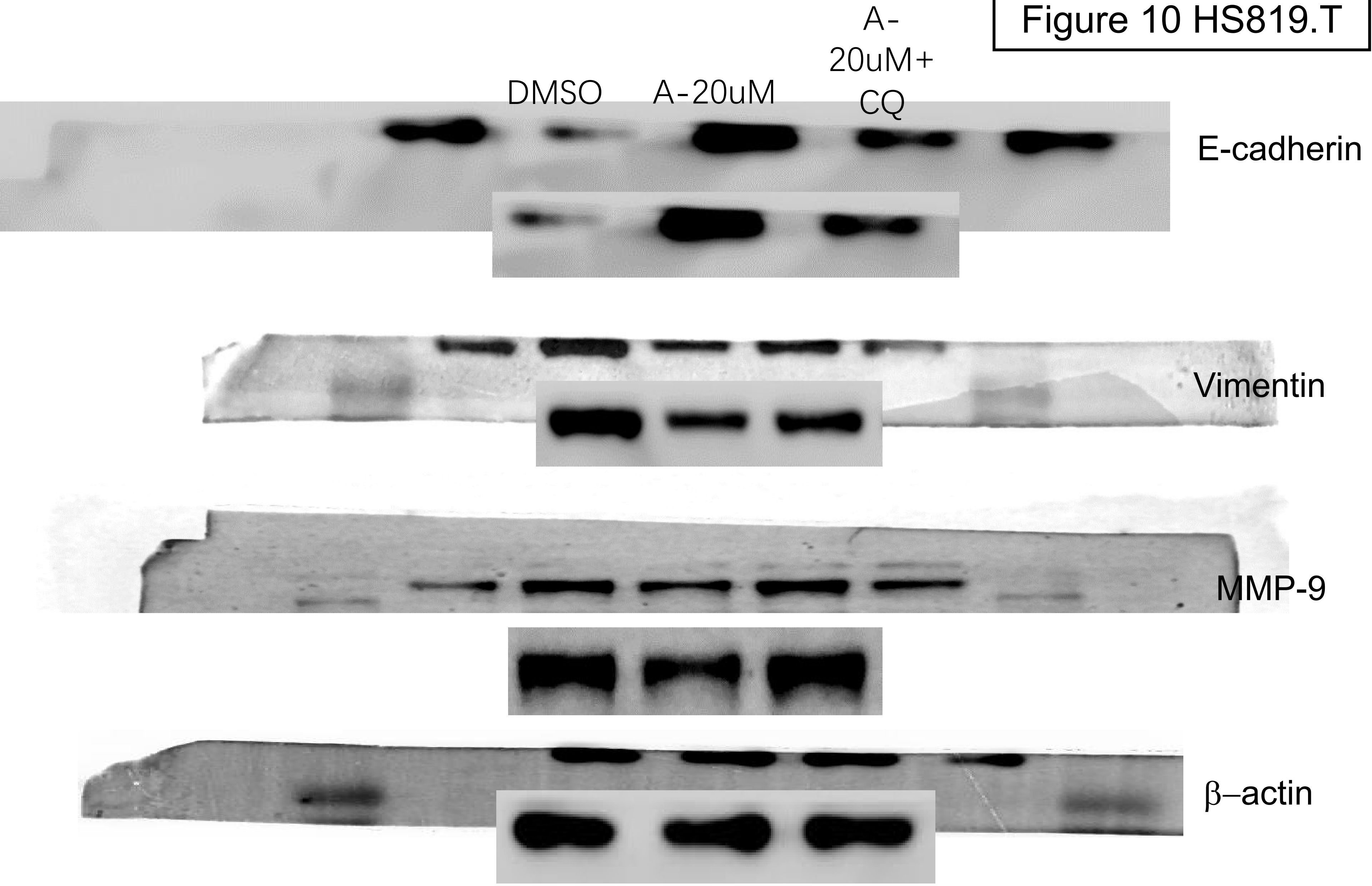

Supplementary Figure 11 HS819.T

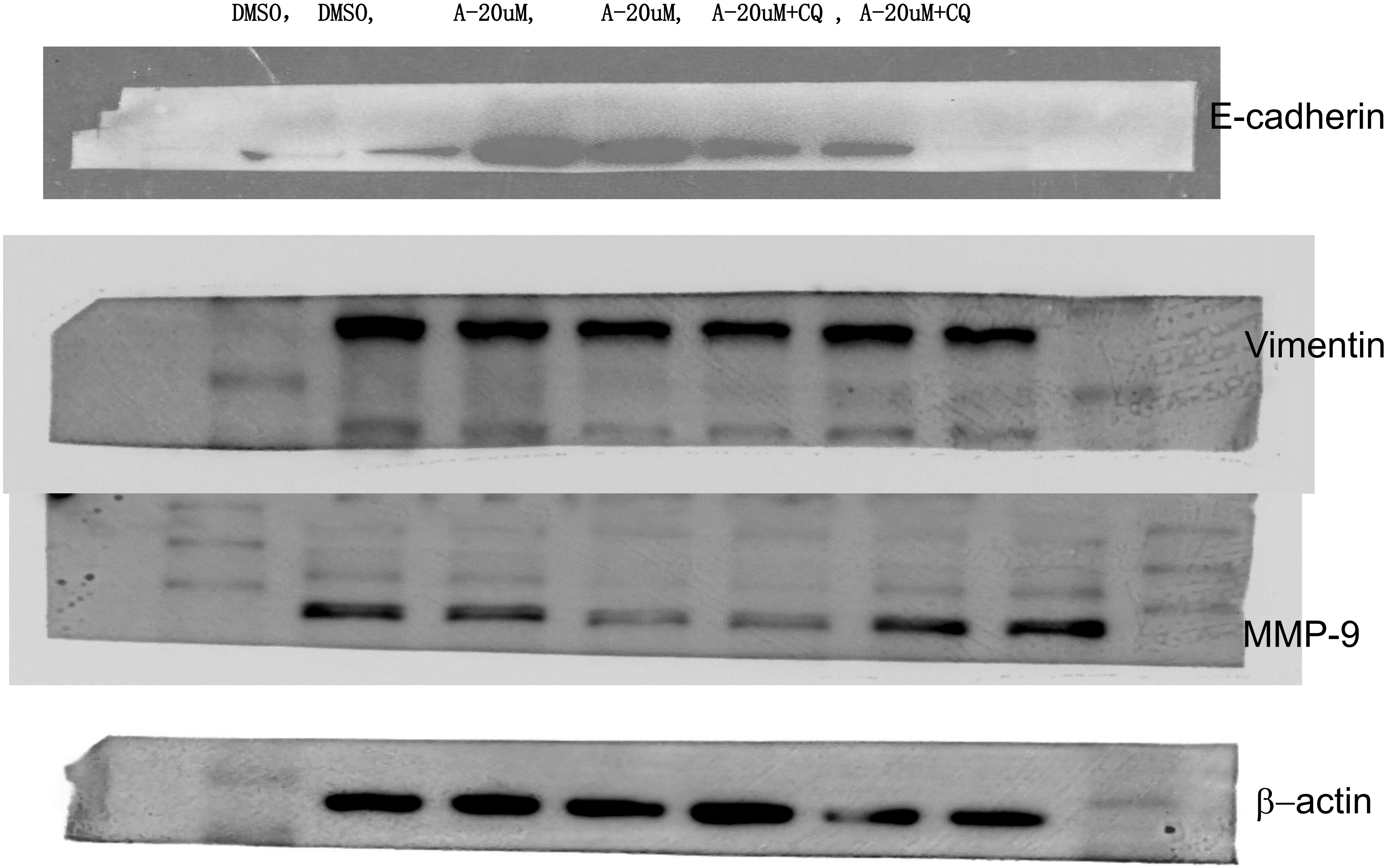

Supplement: Supplementary file 1 [file DataSheet1.pdf]
